# Comparison of Resuscitation Quality in Simulated Pediatric and Adult Out-of-Hospital Cardiac Arrest

**DOI:** 10.1001/jamanetworkopen.2023.13969

**Published:** 2023-05-17

**Authors:** Matthew Hansen, Grace Walker-Stevenson, Nathan Bahr, Tabria Harrod, Garth Meckler, Carl Eriksson, Jeanne-Marie Guise

**Affiliations:** 1 Department of Emergency Medicine, Oregon Health and Science University, Portland; 2 Department of OB/GYN, Oregon Health and Science University, Portland; 3 Departments of Pediatrics and Emergency Medicine, University of British Columbia, Vancouver, British Columbia, Canada; 4 Department of Pediatrics, Oregon Health and Science University, Portland; 5 Department of OB/GYN, Harvard University, Boston, Massachusetts

## Abstract

**Importance:**

Mortality from pediatric out-of-hospital cardiac arrest (OHCA) is high and has not improved in decades, unlike adult mortality. The low frequency of pediatric OHCA and weight-based medication and equipment needs may lead to lower quality of pediatric resuscitation compared with adults.

**Objective:**

To compare the quality of pediatric and adult resuscitation from OHCA in a controlled simulation environment and to evaluate whether teamwork, knowledge, experience, and cognitive load are associated with resuscitation performance.

**Design, Setting, and Participants:**

This cross-sectional in-situ simulation study was conducted between September 2020 and August 2021 in the metropolitan area of Portland, Oregon, and included engine companies from fire-based emergency services (EMS) agencies.

**Exposures:**

Participating EMS crews completed 4 simulation scenarios presented in random order: (1) adult female with ventricular fibrillation; (2) adult female with pulseless electrical activity; (3) school-aged child with ventricular fibrillation; and (4) infant with pulseless electrical activity. All patients were pulseless on EMS arrival. Data were captured by the research team in real time during the scenarios.

**Main outcomes and measures:**

The primary outcome was defect-free care, which included correct cardiopulmonary resuscitation depth, rate, and compression to ventilation ratio, time to bag-mask ventilation, and time to defibrillation, if applicable. Outcomes were determined by direct observation by an experienced physician. Secondary outcomes included additional time-based interventions and the use of correct medication doses and equipment size. We measured teamwork using the clinical teamwork scale, cognitive load with the National Aeronautics and Space Administration task load index (NASA-TLX), and knowledge using advanced life support resuscitation tests.

**Results:**

Among the 215 clinicians (39 crews) who participated in 156 simulations, 200 (93%) were male, and the mean (SD) age was 38.7 (0.6) years. No pediatric shockable scenario was defect free and only 5 pediatric nonshockable scenarios (12.8%) were defect free, while 11 (28.2%) adult shockable scenarios and 27 adult nonshockable scenarios (69.2%) were defect free. The mental demand subscale of the NASA-TLX was higher in the pediatric compared with the adult scenarios (mean [SD] pediatric score, 59.1 [20.7]; mean [SD] adult score, 51.4 [21.1]; *P* = .01). Teamwork scores were not associated with defect-free care.

**Conclusions and Relevance:**

In this simulation study of OHCA, resuscitation quality was significantly lower for pediatric than adult resuscitation. Mental demand may have been a contributor.

## Key Points

**Question:**

Is the quality of prehospital resuscitation in out-of-hospital cardiac arrest different between adults and children?

**Findings:**

In this cross-sectional simulation study of 39 emergency medical services teams, time to specific key interventions was longer for the pediatric cases, and there were more errors in care. Pediatric cases also were noted to have higher mental demand on the National Aeronautics and Space Administration task load index.

**Meaning:**

These findings suggest that resuscitation quality for pediatric out-of-hospital cardiac arrest is lower when compared with adults, and higher cognitive load may be a contributor.

## Teaser

This cross-sectional simulation study compares the quality of pediatric and adult resuscitation from out-of-hospital cardiac arrest (OHCA) in a controlled environment and evaluates whether teamwork, knowledge, experience, and cognitive load are associated with resuscitation performance.

## Introduction

Pediatric out-of-hospital cardiac arrest (OHCA) is a rare but devastating condition that affects 15 000 to 23 000 children annually and represents approximately 15 per 1000 pediatric emergency medical services (EMS) responses.^1,2,3^ Overall, pediatric responses account for 7% to 10% of total EMS responses.^4,5^ Pediatric OHCA cases are particularly challenging for EMS agencies given they are rare, high-stakes, and associated with a significant emotional burden on responders.^6^ Survival from pediatric OHCA has remained around 10% for the last several decades despite improvement in adult outcomes, which may have improved due to an emphasis on cardiopulmonary resuscitation (CPR) quality and high-performance resuscitation of adult patients with OHCA by EMS professionals.^2^

In contrast to adult OHCA resuscitation, previous studies have raised concerns regarding the quality of CPR, timelines of critical actions during pediatric OHCA, medication delivery, and airway management.^7,8,9^ Pediatric arrests are potentially more complex to manage given the need to adapt CPR and airway techniques, equipment size, and medication dose to patient size and weight. This raises the hypothesis that the lack of improvement in pediatric outcomes could be due to lower quality of resuscitative care. However, there have been few studies that directly compare the quality of adult and pediatric OHCA resuscitation. The objective of this study is to evaluate the quality of resuscitation in adult and pediatric shockable and nonshockable OHCA using in situ high fidelity simulation among EMS professionals and evaluate the impact of cognitive load and knowledge on resuscitation quality. We hypothesized that resuscitation quality would be lower in pediatric scenarios than adults, and we would identify specific targets for resuscitative quality improvement.

## Methods

This cross-sectional study was approved by the Oregon Health and Science University institutional review board, and written consent was obtained. This study follows the Strengthening the Reporting of Observational Studies in Epidemiology (STROBE) reporting guideline.^11^

### Study Design, Setting, and Participants

This cross-sectional simulation study was carried out among 7 urban EMS agencies in the Portland metropolitan area spanning 3 counties. This region's EMS system includes public fire and private transport responses for all 911 EMS activations. All agencies are Advanced-Life-Support capable. Fire units typically respond with 3 to 4 firefighters with at least 1 paramedic, and transport agencies have 2 responders with at least 1 paramedic. Portland has relatively high cardiac arrest survival rates, and many agencies have participated in prior cardiac arrest trials.^10^ Due to training restrictions caused by COVID-19, fire and transport agencies were not allowed to train together, so this study was conducted only among the fire agencies. Simulations were completed between September 2020 and August 2021.

Each participating EMS crew completed 4 simulation scenarios that were presented in random order: (1) an adult female with shockable arrest and ventricular fibrillation on arrival; (2) and adult female with pulseless electrical activity; (3) a school aged child with ventricular fibrillation; and (4) an infant with pulseless electrical activity. All patients were pulseless on EMS arrival. Randomization was performed using the Microsoft Excel RANDBETWEEN function. The adult simulator was the Laerdal Resusci-Anne, the school-aged child was the Laerdal SimJunior (45 inches long), and the infant was the Gaumard Newborn Hal (21 inches long). We used an infant simulator for the pediatric nonshockable scenario since we hypothesized that the smallest patients would be the most challenging. Shockable arrests are uncommon in infants; thus we selected a larger pediatric simulator for the shockable arrest scenario after feedback from EMS personnel. A trained actor played the family member for each scenario. Crews were dispatched by radio and responded in an area of the fire station that was set up to look like a home environment. All agencies used their own equipment and equipment kits for the scenarios.

### Study Measurements and Outcomes

Several assessment instruments were used for each simulation scenario. Prior to participation in the simulations, each participant completed a survey that included demographic information. Race and ethnicity data were self-reported. Participants could select American Indian or Alaska Native, Asian, Black, Native Hawaiian or Pacific Islander, White, and Other as race categories. They could also select whether they were of Hispanic or Latino ethnicity. Race data were collected to allow comparison to other studies and local populations, as well as for federally funded research requirements. Paramedics completed an advanced life support knowledge assessment that included questions from the American Heart Association’s adult and pediatric advanced life support knowledge assessments. Each knowledge assessment had 10 questions with scores reflecting the number of correct questions. Potential questions were included based on review by our research team and medical directors of the participating agencies. We included 10 questions to facilitate scoring and ensure the test was short enough to be feasible in the allotted time.

One physician team member (ie, pediatric emergency medicine or critical care) completed a structured technical performance assessment in real-time into a REDCap database during each simulation. This included timestamps for critical interventions, including initiation of CPR, ventilation, vascular access, and medications. Ventilation and CPR quality were also recorded. We established and trained observers on the standards for ventilation and CPR quality. We considered the correct ventilation volume to be squeezing the bag 20% to 50% of its size. This was calculated based on appropriate tidal volumes for each size patient and resuscitation bag volumes. In previous work, we found that video review of cardiac arrest simulation scenarios did not allow for the appropriate visual detail or fields of view to completely capture the data required so we used real-time data capture by a member of the research team who directly observed each scenario. Two research team members completed the Clinical Teamwork Scale for each scenario and then reviewed scores immediately after the scenario to resolve any discrepancies by consensus.^12^ All observers were trained on each assessment tool and had more than 80% percent agreement between selected instrument elements before starting the study using previously collected videos. Study participants also completed the National Aeronautics and Space Administration task load index (NASA-TLX) self-report after each scenario. The NASA-TLX is a validated measure of cognitive load, which evaluates mental demand, physical demand, temporal demand, performance, effort, and frustration and has a range of 0 to 100.^13^ The knowledge and NASA-TLX scores used for analysis came from the paramedic-in-charge who was the same for all 4 scenarios for each team.

We developed definitions of resuscitation errors by extrapolating from Pediatric Advanced Life Support guidelines using the consensus of our team, including experts in pediatric emergency medicine, EMS, pediatric critical care medicine, and patient safety (eTable in the Supplement). Our group of experts reviewed the existing literature and selected these metrics because they were likely to influence OHCA survival.

We compared the rates of errors between the adult and pediatric scenarios. We also developed a definition of defect-free care, which included correct CPR depth (one-third chest depth), rate (100-120 compressions/min), compression-to-ventilation ratio (30:2 adult, 15:2 pediatric), timely bag-mask ventilation (BVM) (started in <120 seconds), and timely defibrillation (<120 seconds) if applicable. For CPR depth, rate, and ratio, we recorded what was used during the majority of the scenario, since variations sometimes were present within scenarios. These elements were selected due to their importance as immediate critical actions in resuscitative care. Defect-free care has been used to describe and compare the overall quality of care provided, especially in scenarios where guidelines are available to direct care, including in cardiac arrest research.^14,15,16,17^

### Statistical Analysis

We first evaluated the participants' demographics using descriptive statistics, including the results of the knowledge assessment for adult and pediatric questions. Next, we summarized overall performance in the simulations, including the time to specific critical actions, including time to start chest compressions, time to first assisted ventilation, time to first defibrillation (for shockable scenarios), and time to the initial dose of epinephrine. We also specifically summarized adult and pediatric CPR performance regarding rate, depth, and technique and compared overall performance using a χ^2^ test. We evaluated the number of errors by domain in each of the scenarios. We also evaluated defect-free care. We compared the NASA-TLX score recorded by the paramedic leading the simulation scenario and Clinical Teamwork Scale (CTS) scores between scenarios using analysis of variance (ANOVA).

We developed a multivariable model to evaluate the association of the variables of interest with defect-free care. Due to the clustering and the fact that no pediatric shockable cases were defect free, we used a bayesian logistic regression approach with the addition of a bayesian shrinkage estimator (1 dummy team with defects in every case and 1 dummy team with no defects in care).^18^ The assumptions of the regression were checked and satisfied for distribution normalcy. The independent variable was the simulation type, and the dependent variable was the defect-free care score. Covariates were selected based on a priori hypotheses of what would influence performance. The model controlled for clustering by the EMS crew. We did not control for clustering within the team because the clinician-level variables were all related to the paramedic in charge rather than the entire team. A subanalysis was performed measuring the association between mental demand and the simulation scenario type to assist in interpreting the primary regression results.

Calculations were performed using Stata IC version 15 (StataCorp). *P* values were calculated with 2-sided *t* tests, and statistical significance was set at *P* < .05. Analyses were performed from July to August 2022.

## Results

### Study Participants

Among the 215 EMS clinicians from 39 crews who participated in the study, 200 (93%) were male, and the mean (SD) age was 38.7 (0.6) years, reflecting the general demographics of the firefighting workforce. Table 1 displays demographic information for study participants. More than half of the participants chose not to identify their race, though among those who did respond, the large majority were White individuals. Participants had a mean (SD) of 10 (4.1) years of experience in EMS.

**Table 1.** Participant Demographics

Characteristics	EMTs and paramedics, No. (%), n = 215
Age, mean (SD), years	38.7 (0.6)
Sex	
Female	15 (7.0)
Male	200 (93.0)
Race	
Did not indicate	113 (52.6)
Black or African American	3 (1.4)
Hawaiian or Pacific Islander	0
Native American	2 (1.0)
White	96 (44.7)
Other^a^	5 (2.3)
Total years of experience, mean (SD)	9.9 (4.1)
Years at current level of training, mean (SD)	9.9 (6.9)
Knowledge assessment score adult, mean (SD)	8.6 (1.1)
Knowledge assessment score pediatric, mean (SD)	8.2 (1.3)

^a^ Other includes Asian individuals or generic selction of other in the responses.

### Resuscitation Quality

Table 2 displays aggregated data for several cardiac arrest resuscitation performance metrics from the simulations. We noted that in both pediatric scenarios, there were delays in time to CPR, time to BVM, vascular access, and time to the initial dose of epinephrine relative to adults. The greatest delays were observed in the pediatric nonshockable scenario using the infant simulator. Twenty-four nonshockable infant cases (61.5%) and 21 shockable pediatric cases (53.8%) used the correct 15:2 compression-to-ventilation ratio. Incorrect pediatric compression-to-ventilation ratios observed included continuous compressions, 30:2, and 3:1. All adult cases used either continuous chest compressions or 30:2, and 21 of 78 pediatric cases (27.0%) had more than 120 compressions per minute.

**Table 2.** Cardiac Arrest Resuscitation Performance Metrics From 4 Simulation Scenarios

Metrics	Patient, No. (%)			
	Adult nonshockable arrest (n = 39)	Adult shockable arrest (n = 39)	Pediatric nonshockable arrest (n = 39)	Pediatric shockable arrest (n = 39)
Time to first CPR, mean (SD), minutes	0.2 (0.1)	0.3 (0.1)	0.6 (0.3)	0.4 (0.6)
Time to first BVM, mean (SD), minutes	1.3 (0.6)	1.3 (0.6)	1.5 (0.9)	1.4 (0.6)
Time to vascular access, mean (SD), minutes	2.6 (0.8)	3.0 (1.0)	3.7 (1.6)	3.3 (1.3)
Time to first epinephrine dose, mean (SD), minutes	3.9 (1.0)	4.3 (1.4)	5.3 (1.2)	4.9 (1.5)
Time to first advanced airway attempt, mean (SD), minutes	4.8 (1.7)	5.0 (3.1)	6.0 (1.9)	5.4 (1.7)
Mean No. of intubation attempts, mean (SD), minutes	0.9 (0.4)	1.0 (0.4)	1.3 (1.1)	1.0 (1.1)
Cases where SGA used	2 (5.1)	1 (2.5)	3 (7.7)	5 (12.8)
Compression/ventilation ratios by percentage				
Continuous	13 (33.3)	14 (35.9)	8 (20.5)	11 (28.2)
30:2	22 (56.4)	23 (59.0)	3 (7.7)	7 (17.9)
15:2	0	0	24 (61.5)	21 (53.8)
3:1	0	0	4 (10.3)	0
Mechanical CPR	4 (10.3)	2 (5.1)	0	0
Compression rate (majority of the time) by percentage				
<80	0	2 (5.1)	1 (2.6)	1 (2.6)
88-99	2 (5.1)	1 (2.6)	4 (10.3)	2 (5.1)
100-120	33 (84.6)	31 (79.5)	24 (61.5)	25 (64.1)
>120	4 (10.3)	5 (12.8)	10 (25.6)	11 (28.2)
CPR technique by percentage				
Mechanical				
2 hands	39 (100.0)	39 (100.0)	0	25 (64.1)
1 hand	0	0	0	14 (35.9)
2 fingers	0	0	26 (66.7)	0
2 hands encircling	0	0	13 (33.3)	0
CPR depth sufficient	38 (97.4)	38 (97.4)	14 (35.9)	19 (48.7)
Postsimulation assessments, mean (SD) score				
NASA-TLX total	39 (13)	40 (14)	47 (14)	43 (15)
NASA-TLX mental demand	46 (22)	54 (19)	61 (20)	57 (21)
CTS teamwork	8 (1)	8 (1)	8 (1)	8 (1)
Defect-free care	27 (69.2)	11 (28.2)	5 (12.8)	0

Abbreviations: BVM, bag-mask ventilation; CPR, cardiopulmonary resuscitation; NASA-TLX, National Aeronautics and Space Administration task load index.

Table 3 compares the frequency of errors in the adult and pediatric simulation scenarios. Generally, there were more errors in the pediatric scenarios. An incorrect mask size (too large) was used in 43 pediatric scenarios (55.1%) and only 2 adult scenarios (2.6%). The CPR depth was too shallow in 45 pediatric cases (57.7%) and only 2 adult scenarios (2.6%). While there were no epinephrine or defibrillation dosing errors in adults, there were dosing errors in medication (16 [20.5%]) and electricity (15 [19.2%]) in pediatric scenarios, and all of these were overdoses.

**Table 3.** Comparison of Errors Between Adult and Pediatric Simulations^a^

Error type	Simulations, No. (%)	
	Adult (n = 78)	Pediatric (n = 78)
Delay in pulse check >60 s	1 (1.3)	3 (3.8)
Delay in defibrillation >120 s for shockable simulations (n = 39)	21 (53.8)	31 (79.4)
Delay in starting PPV >120 s	11 (14.1)	14 (17.9)
Incorrect mask used for BVM	2 (2.6)	43 (55.1)
Delay in starting CPR >60 s	0	5 (6.4)
Continuous compressions before advanced airway	27 (34.6)	16 (20.5)
CPR depth too shallow or too deep	2 (2.6)	45 (57.7)
>2 attempts at advanced airway	0	9 (11.5)
EtCO_2_ not used to confirm airway within 30 s	30 (38.5)	45 (57.7)
ETT size incorrect^b^	11 (14.1)	20 (25.6)
Breath volume <20% or >50% of bag volume	37 (47.4)	39 (50.0)
CPR rate >120 or <100	18 (23.1)	31 (39.7)
Incorrect CPR ratio^c^	0	25 (32.1)
IV/IO not done in 10 min	0	0
Epinephrine not given in 10 min	0	5 (6.4)
Epinephrine dosing error	0	16 (20.5)
Defibrillation electricity dosing error	0	15 (19.2)
Total	160 (12.4)	362 (28.1)

Abbreviations: BVM, bag-mask ventilation; CPR, cardiopulmonary resuscitation; EtCO_2_, end-tidal carbon dioxide; ETT, endotracheal tube; PPV, pulse pressure variation.

^a^ χ^2^ comparison of the overall proportions: *P* < .01.

^b^ Correct sizes: adults, 7-8; child, 4.5-5; infant, 3.0.

^c^ Correct ratio: adults, 30:2; child, 15:2; infant, 15:2 or 3:1.

### Defect-Free Resuscitation

The Figure displays the number of defects and the number of cases with defect-free care among the 4 simulation scenarios. Of note, none of the pediatric shockable scenarios and only 5 pediatric nonshockable scenarios (12.8%) were defect free, while 27 adult nonshockable scenarios (69.2%) were defect free.

**Figure.**
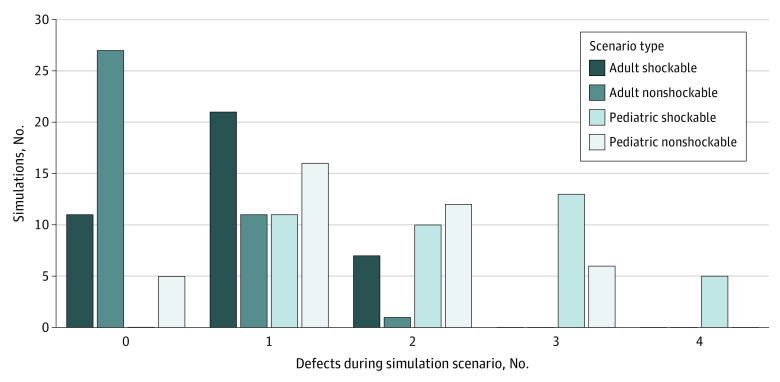
Number of Major Defects in Care by Simulation Scenario Type

### Cognitive Load

We compared the mean (SD) NASA-TLX scores of the paramedic team leader for each scenario. The highest cognitive load was observed for the pediatric nonshockable scenario (48.4 [13.8]) followed next by the pediatric shockable scenario (43.6 [15.2]), adult shockable (41.1 [13.7]), and adult nonshockable scenario (39.3 [13.3]).

The overall mean (SD) NASA-TLX scores were significantly higher in the pediatric compared with the adult scenarios using 1-way ANOVA (pediatric, 45.4 [14.4]; adult, 41.2 [13.5]; *P* = .01). Similarly, the mental demand subscale of the mean (SD) NASA-TLX was higher in the pediatric compared with the adult scenarios (pediatric, 59.1 [20.7]; adult, 51.4 [21.1]; *P* = .01). The lowest mean (SD) CTS was observed in the pediatric nonshockable scenario (6.6 [1.6]). The highest mean (SD) CTS was from the adult nonshockable arrest (7.4 [1.4]). The difference in overall CTS among the 4 scenarios was not statistically significant using ANOVA with *P* = .062 and was relatively small in size. Overall knowledge test scores were not significantly different between the adult and pediatric knowledge assessments.

### Multivariable Analysis

In the multivariable model, the posterior probability of defect-free care was highly associated with scenario type. We noted probabilities of defect-free care of 67% (95% CI, 50% to 84%), 29% (95% CI, 15% to 44%), 17% (95% CI, 5% to 28%) and 3% (95% CI, −3% to 8%) for the adult nonshockable, adult shockable, pediatric nonshockable, and pediatric shockable scenarios, respectively. Table 4 displays the regression results. In an additional analysis, the mental demand was associated with the scenario type. The pediatric nonshockable scenario was associated with a 10.6-point increase in mental demand (95% CI, 6.3 to 15.0 points). An increase in the Pediatric Advanced Life Support, or PALS, knowledge assessment score was associated with a 5% higher probability of defect-free care (95% CI, 0.6%-10.3%). The clinical teamwork scale, NASA-TLX score, years of experience, and ACLS knowledge evaluation score were not associated with defect-free care.

**Table 4.** Multivariable Regression Results

Variable	Posterior probability (95% CI)
Simulation scenario	
Pediatric nonshockable scenario	0.26 (−0.025 to 0.077)
Pediatric shockable scenario	0.17 (0.49 to 0.28)
Adult nonshockable scenario	0.67 (0.50 to 0.84)
Adult shockable scenario	0.29 (0.15 to 0.44)
Other variables^a^	
CTS Teamwork score	0.002 (−0.045 to 0.050)
NASA-TLX mental demand score	−0.003 (−0.007 to 0.001)
Team leader years of experience	−0.001 (−0.010 to −0.10)
PALS knowledge score	0.054 (0.006 to 0.10)
ACLS knowledge score	−0.006 (−0.052 to 0.041)

Abbreviations: ACLS, Advanced Cardiac Life Support; CTS, clinical teamwork score; NASA-TLX, National Aeronautics and Space Administration task load index; PALS, Pediatric Advanced Life Support.

^a^ Data are presented as marginal effects (95% CI).

## Discussion

In this simulation-based study comparing pediatric and adult OHCA care by EMS agencies in an urban area, we found that resuscitation performance was of lower quality and associated with higher cognitive load in pediatric scenarios compared with adult scenarios, while resuscitation knowledge was similar. Differences in care that are likely clinically important include inadequate chest compression depth in 58% of pediatric cases and chest compression rate of more than 120 in 27% of pediatric cases. Adult resuscitation quality was better than pediatric, but there was room for improvement in time to defibrillation and using the correct BVM volume. We noted a statistically significant difference in teamwork, but the difference was small and of unclear clinical relevance. The greatest challenges were encountered in the pediatric nonshockable scenario, which used the infant simulator. High mental demand was a primary driver of the increase in cognitive load and was also highest in the scenario with the smallest simulator. Multivariable modeling showed the specific scenario was the most substantial factor associated with defect-free care. Mental demand was highly correlated with the scenario type, which could be why it was not significant in the multivariable model. This study is novel by directly comparing adult care with pediatric care in a controlled environment and shows that high cognitive load is a potential root cause of resuscitation performance deficits in pediatric care compared with adult care and may be more important than resuscitation algorithm knowledge or teamwork. Efforts to improve cognitive load may improve the quality of pediatric OHCA resuscitation.

Several studies have evaluated the safety and quality of care provided during pediatric OHCA. One chart review study found that about 30% of cases were complicated by a serious adverse event, with medication overdoses and airway management errors as the most common.^9^ Another study evaluated the timeliness of care in simulated OHCA and found significant delays in the initiation of positive pressure ventilation and epinephrine administration though the thresholds for considering a delay were different. Another study compared adult and pediatric cardiac arrest outcomes for arrests that took place in the emergency department and found no significant differences in process measure or outcomes, though this is not likely generalizable to the EMS setting.^19^ To our knowledge, this is the first direct comparison of adult and pediatric OHCA resuscitation quality.

There are many potential reasons why the cognitive load was higher in the pediatric simulation scenarios. All pediatric medications need to be dosed based on the patient’s length, and equipment needs to be selected based on the patient’s size. In contrast, medication doses in adults are all the same, and there is little variability in equipment size requirements. The smaller pediatric patient size could lead to crowding to gain access to the patient and less physical space to work in, resulting in more stress. In this EMS system, all agencies use a locally developed length-based resuscitation guide that includes all medications and equipment. There are also differences in the pediatric resuscitation algorithms, including the compression-to-ventilation ratios and CPR techniques, which may increase cognitive load due to less muscle memory compared with adults. Finally, there is likely an emotional component as well. Previous studies have shown that paramedics sympathize, feel stress due to the innocence of children, and may experience stress from family or other bystanders.^6^ In simulations, the emotional toll is likely less than in clinical practice. However, we had trained actors playing family members to increase fidelity and did note CPR depth was insufficient in children suggesting paramedics inherently may be hesitant or fear causing harm.

One potential solution to improve pediatric resuscitation quality could be an improved cognitive aid for pediatric resuscitation. Currently, we are not aware of a cognitive aid that incorporates both what is supposed to be done with how it is to be done. Paramedics typically use a specific cognitive aid for medications and equipment (how), though it does not usually provide guidance on the algorithm of care (what). Combining these elements in a user-friendly platform may be a promising solution since it could reduce the cognitive load on the team. Further research should evaluate the efficacy of cognitive aids as well as investigate specific tasks or procedures that may be associated with the highest cognitive load. It is also possible that a team could more effectively distribute cognitive load among team members. In this study, we only assessed the cognitive load of the team leader. Simplifying care could also potentially reduce cognitive load. Most participants had advanced airway management with an endotracheal tube, while a supraglottic airway device or bag-mask-ventilation is simpler and may reduce cognitive load.

### Limitations

This study has several limitations. First, all events were simulated and cannot be correlated with clinical outcomes. Therefore, findings may not be generalizable true resuscitation quality. Using simulation was necessary due to the rare nature of pediatric OHCA cases. Furthermore, simulation allows detailed data capture that is not feasible in real time. Simulated performance is likely better than real life due to the more controlled environment and lower stress so this likely biases our results in favor of better overall performance than what would be seen in a clinical setting. This study was conducted in a single geographic area with relatively high OHCA survival and may not generalize to other EMS systems. Our sample size was also modest, limiting our ability to perform multivariable analyses.

## Conclusions

In this study of simulated pediatric and adult OHCA, pediatric cases consistently had delays in initiating critical interventions and lower-quality care, while paramedics also experienced significantly higher cognitive load in the pediatric scenarios. Increased cognitive load may contribute to challenges in pediatric resuscitation.

## Notes

Supplement 1. **eTable.** Simulation Error Assessment for Pediatrics and Adult Simulations

Supplement 2.
Data Sharing Statement
