# Supplementary material for: Comparison of Resuscitation Quality in Simulated Pediatric and Adult Out-of-Hospital Cardiac Arrest
Source: JAMA Netw Open. 2023 May 17;6(5):e2313969. doi: 10.1001/jamanetworkopen.2023.13969 (PMC10193176; doi:10.1001/jamanetworkopen.2023.13969)
Supplement: Supplement 1. — eTable. Simulation Error Assessment for Pediatrics and Adult Simulations [file jamanetwopen-e2313969-s001.pdf]

Supplemental Online Content

Hansen M, Walker-Stevenson G, Bahr N, et al. Comparison of resuscitation quality in simulated pediatric and adult out-of-hospital cardiac arrest. *JAMA Netw Open.* 2023;6(5):e2313969. doi:10.1001/jamanetworkopen.2023.13969

eTable. Simulation Error Assessment for Pediatrics and Adult Simulations

This supplemental material has been provided by the authors to give readers additional information about their work.

© 2023 Hansen M et al. *JAMA Network Open.*

eTable. Simulation Error Assessment for Pediatrics and Adult Simulations

Event	Pediatric Error (non infant)	Adult Error	Rationale
Breathing assessment timeliness	Not Done or > 60 secs	Not Done or > 60 secs	AHA says immediately. Expert panel judgement for timing
Pulse timeliness	Not Done or > 60 secs	Not Done or > 60 secs	AHA says immediately. Expert panel judgement for timing
Pulse check duration	<5 or >10 seconds	<5 or >10 seconds	AHA guidance
Rhythm Check	> 2 minutes	> 2 minutes	AHA guidance- 2 mins CPR then rhythm check
PPV timeliness	Not Done or > 120 secs		120 secs- Expert panel judgement. Not done- PALS guidance. AHA COIVD response- compression only for adult
PPV equipment	Incorrect size BVM	Incorrect size BVM	Expert panel judgement
CPR timeliness	Not Done or > 60 secs	Not Done or > 60 secs	AHA says immediately after pulse check. Expert panel judgement for timing
Compression:ventilation Ratio	Anything other than 15:2	Neither 30:2 or continuous	AHA guidance
CPR rate	>120 or <100	>120 or <100	AHA guidance
CPR rotation	>2 minutes between rotation or indicated as infrequent by reviewer	>2 minutes between rotation or indicated as infrequent by reviewer	AHA guidance<br>*might not have collected comprehensive data on this
CPR Depth	Less than 1/3 of AP chest diameter	Less than 2 in (5cm)	AHA guidance
Measure length/ Determined weight	Not Done	**N/A**	AHA guidance<br>*might not have collected comprehensive data on this
Advanced airway	>2 attempts	>2 attempts	Expert panel judgement
Confirm advanced airway	ETC02 not done within 30 seconds	ETC02 not done within 30 seconds	AHA recommends capnography. Expert panel judgement for timing. (REDCap checkbox)
ETT size	Infant: <3.0, >4.0<br>Jr: <4.5, >5.5	<6.5,>7.5	Expert panel judgement
ETT Depth	Indicated incorrect by reviewer<br>• Too deep<br>• Too shallow	Indicated incorrect by reviewer<br>• Too deep<br>• Too shallow	Expert panel judgement
Breath Volume	<20% or >50%	<20% or >50%	Expert panel judgement
~~Bag size~~	~~Incorrect size BVM~~	~~N/A~~	

© 2023 Hansen M et al. *JAMA Network Open.*

IO/IV	Not Done or > 10 min	Not Done or > 10 min	PALS states to establish IV/IO access. Expert panel judgement for timing (based on needs for vascular access for time based drugs)
Epinephrine timeliness	Not Done or > 10 min	Not Done or > 10 min	AHA states ASAP for NS and after 2nd shock for shockable. Expert panel judgement for time (peds data showed improved survival for early administration)
Epinephrine dosing	Not Done OR<br>Infant: <0.3 or >0.5 mL (0.03-0.05 mg)<br>Child: <2.2. or >2.6 mL (0.22-0.26 mg)	Dose other than 1 mg	AHA guidelines. Expert panel judgement for acceptable margin of error
1st Defib Dose	Not Done OR<br>Infant: <6.4j or >19.2j<br>Child: <35j or >106j	<120 >360 joules	AHA guidelines- adult is 120-200 joules (need to confirm based on manufacturer). 4kg infant weight based on CDC table for 21 inches. 22kg Sim Jr. Range = 2-4 j/kg +/- 20%.

© 2023 Hansen M et al. *JAMA Network Open.*
