# Supplementary material for: Comparison of Resuscitation Quality in Simulated Pediatric and Adult Out-of-Hospital Cardiac Arrest
Source: JAMA Netw Open. 2023 May 17;6(5):e2313969. doi: 10.1001/jamanetworkopen.2023.13969 (PMC10193176; doi:10.1001/jamanetworkopen.2023.13969)
Supplement: Supplement 2. — Data Sharing Statement [file jamanetwopen-e2313969-s002.pdf]

Data Sharing Statement

Hansen. Comparison of Resuscitation Quality in Simulated Pediatric and Adult Out-of-Hospital Cardiac Arrest. *JAMA Netw Open*. Published May 17, 2023.
doi:10.1001/jamanetworkopen.2023.13969

Data
Data available: No
